# Effects of a smartphone application named “Shared Decision Making Assistant” for informed patients with primary liver cancer in decision-making in China: a quasi-experimental study

**DOI:** 10.1186/s12911-022-01883-w

**Published:** 2022-05-31

**Authors:** Sitong Wang, Qingwen Lu, Zhixia Ye, Fang Liu, Ning Yang, Zeya Pan, Yu Li, Li Li

**Affiliations:** 1 Department of Nursing, Eastern Hepatobiliary Surgery Hospital, Naval Medical University, No. 700 Moyu Road, Jiading District, Shanghai, 201805 People’s Republic of China; 2 Department of No. 5 Hepatobiliary Surgery, Eastern Hepatobiliary Surgery Hospital, Naval Medical University, Shanghai, 201805 People’s Republic of China; 3 Department of No. 3 Hepatobiliary Surgery, Eastern Hepatobiliary Surgery Hospital, Naval Medical University, Shanghai, 201805 People’s Republic of China; 4 Department of Organ Transplantation, Eastern Hepatobiliary Surgery Hospital, Naval Medical University, Shanghai, 201805 People’s Republic of China; 5 Officers’ Ward, General Hospital of Northern Theater Command, Shenyang, 110016 Liaoning People’s Republic of China

**Keywords:** Decision aids, Smartphone application, Primary liver cancer, Patient, Decision-making

## Abstract

**Background:**

It is well known that decision aids can promote patients’ participation in decision-making, increase patients’ decision preparation and reduce decision conflict. The goal of this study is to explore the effects of a “Shared Decision Making Assistant” smartphone application on the decision-making of informed patients with Primary Liver Cancer (PLC) in China.

**Methods:**

In this quasi-experimental study , 180 PLC patients who knew their real diagnoses in the Eastern Hepatobiliary Surgery Hospital, Naval Medical University, Shanghai, China, from April to December 2020 were randomly assigned to a control group and an intervention group. Patients in the intervention group had an access to the “Shared Decision Making Assistant” application in decision-making, which included primary liver cancer treatment knowledge, decision aids path, continuing nursing care video clips, latest information browsing and interactive platforms. The study used decision conflict scores to evaluate the primary outcome, and the data of decision preparation, decision self-efficacy, decision satisfaction and regret, and knowledge of PLC treatment for secondary outcomes. Then, the data were entered into the SPSS 22.0 software and were analyzed by descriptive statistics, Chi-square, independent t-test, paired t-test, and Mann–Whitney tests.

**Results:**

Informed PLC patients in the intervention group (“SDM Assistant” group) had significantly lower decision conflict scores than those in the control group. (“SDM Assistant” group: 16.89 ± 8.80 vs. control group: 26.75 ± 9.79, *P* < 0.05). Meanwhile, the decision preparation score (80.73 ± 8.16), decision self-efficacy score (87.75 ± 6.87), decision satisfaction score (25.68 ± 2.10) and knowledge of PLC treatment score (14.52 ± 1.91) of the intervention group were significantly higher than those of the control group patients (*P* < 0.05) at the end of the study. However, the scores of “regret of decision making” between the two groups had no statistical significance after 3 months (*P* > 0.05).

**Conclusions:**

Access to the “Shared Decision Making Assistant” enhanced the PLC patients’ performance and improved their quality of decision making in the areas of decision conflict, decision preparation, decision self-efficacy, knowledge of PLC treatment and satisfaction. Therefore, we recommend promoting and updating the “Shared Decision Making Assistant” in clinical employment and future studies.

## Background

Primary liver cancer (PLC), one of the most common cancers, is the fourth most lethal tumor and ranks sixth in terms of incident cases worldwide [1]. With a higher incidence than a lot of other countries, China is recognized as a country where liver disease is an endemic. Though accounting for only 19% of the global population, China sees more than half of the new PLC cases and deaths [2]. Although the Chinese National Cancer Center (CNCC) estimates that the incidence and mortality rates of PLC are expected to decrease by 2030, the burden of PLC is still quite heavy in China, especially in rural and western areas [3]. Moreover, it cannot be ignored that the treatment of PLC is a long process of dynamic professional clinical exploration and practice, comprised of surgery, ablation, chemotherapy, radiotherapy, immunotherapy, traditional Chinese medicine and other treatment schemes [1]. Faced with such diverse alternative treatment options, patients frequently suffer from distress, fear and uncertainty in decision-making as a result of unequal access to disease diagnosis, treatment and prognosis due to the differences in economy, culture and medical capabilities in different regions of China [4]. Therefore, PLC patients would often passively plunge into the plight of treatment decision-making. And it is especially difficult for patients to determine the unique and optimal therapeutic regime [5, 6].

In China, a survey with 556 informed PLC patients showed that 78.2% of the patients intended to make treatment decisions, but only 21.8% had actually done so. Of those patients who had expressed their wish for decision-making, 48.6% had met “decision conflict”, and 52.4% had reported inadequate information provision. The study suggested that medical staff should provide sufficient information to help PLC patients make appropriate decisions regarding their treatments [7].

Shared decision-making (SDM) between medical staff and patients has been confirmed to be a successful way for helping patients make their own decisions [8]. In SDM field, decision aids (DAs) have been carefully developed that include pamphlets, websites or video clips, as educational tools to provide decision-making information, improve patients’ ability in choosing among various treatment options, as well as complementing physicians’ suggestions [9]. Plenty of studies demonstrated that decision aids could improve patients' cognition of pros and cons in medical decision-making, reduce decision conflict, improve satisfaction with the final decision, and finally improve the quality of medical service [10–13].

Therefore, we speculated that decision aids could provide support in decision-making for informed PLC patients. With the development of information technology, it has been penetrating into many fields of the medical industry, such as appointment registration, drug management and patient self-monitoring [14–16]. Evidence shows that decision aids are effective for cancer patients through mobile applications (apps), for example, the decision aid software Lung Talk [17] promoted lung cancer screening, and other applications assist females in breast self-examination [18]. Unfortunately, there was a dearth of decision aids for PLC patients in China. To address this problem, an interactive PLC patient decision aid smartphone application, called “Shared Decision Making Assistant” (SDM Assistant), was developed using Ottawa Decision Support Framework (ODSF). To our knowledge, this is the first controlled trial with a decision aid application for informed PLC patients. The primary aim of the study was to analyze decision conflict, and the secondary objectives were to explore decision preparation, decision self-efficacy, decision satisfaction and knowledge about PLC, as well as the patients’ decision regret 3 months after discharge.

## Materials and methods

This prospective, single-center, quasi-experimental study was approved by the Institutional Research Ethics Committee of Eastern Hepatobiliary Surgery Hospital, Naval Medical University, Shanghai, China, and a written informed consent was obtained from each subject.

### Design and participants

With a controlled pre-and-post-test design, this study was conducted on the informed PLC patients referred to our hospital from April to December 2020. The change of decision conflict score was an important indicator to evaluate the effect of “SDM Assistant”. Therefore, by reference to a previous study [7] that reported the mean ± standard deviation (SD) of decision conflict in two groups as 20.23 ± 12.99 and 27.13 ± 17.38, α = 0.05 power of 90%, β = 0.20, and according to literature, t_α/2_ = 1.96, t_β_ = 1.283, a 154-subject sample size (n = 76 in each group) was estimated for the research by using the G-power software. Considering a 10% probability of loss, the sample size was increased to 84 individuals in each group. To facilitate calculation, this study further expanded the sample size and a total of 100 informed PLC patients were planned to be included into each group.

Inclusion criteria for informed PLC patients included: (1) preliminarily diagnosed with PLC; (2) diagnosis informed and willing to participate in the study; (3) 18 years old or above; (4) knowing the purpose of the study and consenting to the study; (5) early or mid-stage PLC, which was 0/A/B according to the BCLC, after physician screening; (6) with literacy and digital skills; (7) having smartphones. Exclusion criteria included: (1) Metastatic liver cancer; (2) Serious changes occurred during the trial; (3) The patients or their family members requested to withdraw.

**Fig. 1:**
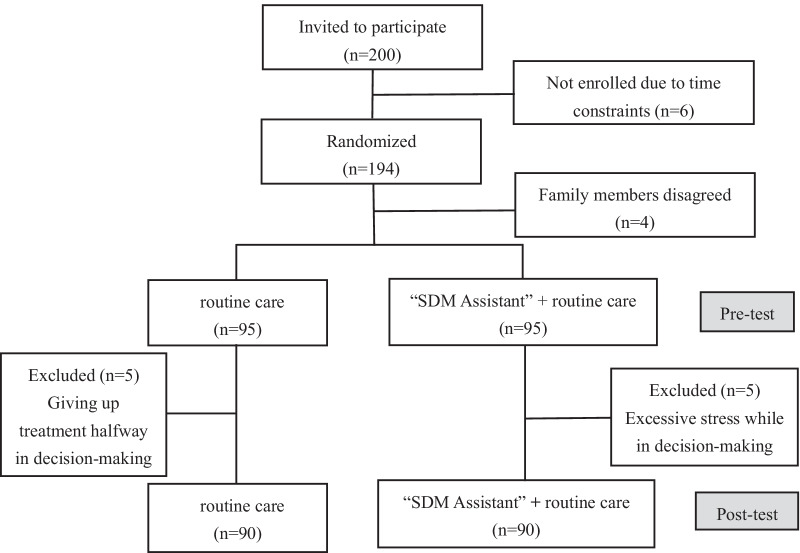
Diagram details patient flow in the trial

This study was conducted by the PLC decision aids team of Eastern Hepatobiliary Surgery Hospital (team members: 8 PLC physicians, 12 hepatobiliary specialist nurses and 2 psychologists). This hospital is the largest specialized hospital in hepatobiliary surgery in the world, with more than 11,000 liver cancer inpatients each year, making data collection easy and research results representative, and at the same time providing sufficient sample size for the research. Totally, 190 informed PLC patients were selected by simple random sampling, using the table of random numbers. Then they were randomly assigned in a 1:1 ratio to 2 groups: cohort I received routine medical care while cohort II downloaded the “SDM Assistant” app. The allocation was based on a computer-generated sequence of random numbers. This study was unblinded for all parties because blinding of the physicians and nurses could not be guaranteed. Shortly after the beginning of the research, 5 individuals in the intervention group were excluded due to excessive stress in decision making process, and another 5 individuals in the control group were also excluded because they gave up treatment halfway in hospital. Thus, the number of participants in the two groups decreased to 90 in each, as is shown in Fig. 1.

### Development of the “Shared Decision Making Assistant” App

First, we conducted individual interviews to identify the specific needs and status of PLC patients participating in shared decision making. Combined with literature review, a decision aids outline was constructed for PLC patients. Then, an expert group discussion was held to scientifically evaluate the outline. After that, two rounds of e-Delphi study finalized the structure and content of “SDM Assistant”. Finally, the materials were arranged in the corresponding modules of the “SDM Assistant” in a diversified means of expression (video, audio, text, 3D image, treatment scheme effect comparison diagram, etc.) with the help of the programming environment for the Android platform, which is called Android Studio.

In all, the “SDM Assistant” included two core parts [19]:“PLC Treatment Knowledge Center” and “Decision Aids Path”. The “PLC Treatment Knowledge Center” includes the epidemiology of PLC and clarification of the 12 PLC treatment schemes, each of which covers the principle, indications, contraindications, preoperative preparation, postoperative care, complications, advantages, disadvantages, subsequent therapy, postoperative recurrent rate and health education after discharge. The language is at a sixth grade reading level. Based on the Ottawa Decision Support Framework, the “Decision Aids Path” includes a total of five steps, designed to help patients clarify the choice of preferred treatment options.

### Intervention

Participants of the two groups were admitted into two wards (on different floors) of the same department. Because of COVID-19 prevention policy, participants could not leave their respective wards. Therefore, they did not interfere with each other. Participants assigned to the intervention group used the “SDM Assistant”, while participants in the control group received standardized medical and nursing procedures of the hospital, and did not use the “SDM Assistant”. The intervention time was from the date of admission to the completion of decision-making, and the participants’ decision regret was evaluated at 3-month follow-up.

After installing the “SDM Assistant” with the help of nurses, participants in the intervention group were required to input their demographic information as well as their disease-related information. Nurses explained how to use the app, and then participants made decisions with the use of “SDM Assistant”. They were asked to contact the researcher in case they had any problem or question regarding working with the application.

Specific process was as follows: first, participants in the intervention group browsed the “PLC Treatment Knowledge Center”, which included PLC epidemiological information, PLC laboratory examination information and information related to 12 treatment schemes of PLC (including surgery, transcatheter arterial chemoembolization, cryoablation therapy, chemoembolization, radiofrequency ablation, microwave ablation, transplantation, percutaneous ethanol injection, targeted therapy, radiation therapy, immunotherapy, chemotherapy, and traditional Chinese medicine treatment). This part provided participants with valuable knowledge about PLC-related treatment. Participants were asked to complete the “Decision Aids Path” afterwards, which consisted of five steps: (1) The participant had a face-to-face conversation with the doctor to determine the treatment options available; (2) Comparing alternative treatment options on app that include principle, therapy pathway, indications, advantages, disadvantages, operation time, postoperative average length of hospitalization, complications, cost and 5-year recurrence rate; this was what patients were most interested in. (3) Exploring the preferences. Participants could score the risks and benefits of different treatment options by using the Likert 5-point scoring system through the app. After selection, the scores of the difference would be calculated between the advantages and disadvantages of each treatment scheme, so that participants could intuitively see the advantages and disadvantages of each treatment scheme, which guided participants to ponder over the choice of treatment scheme; (4) Knowledge testing. After the completion of the first three steps, the participants were asked questions about PLC treatment to determine whether the participants’ choice was actually based on a correct understanding of comprehensive disease treatment knowledge. (5) Under China’s current medical system, the decision-making must be jointly made by doctors and patients. Therefore, after the first four steps, there was another face-to-face conversation between doctors and patients to finalize the treatment plan. Finally, “SDM Assistant” would remind the participants to evaluate and summarize the decision on the following day. The reminder included an alarm ring and a message on top of smartphone. It is noteworthy that the application’s feedback system immediately transfers the participants’ information to the researcher.

### Measurements

#### Sociodemographic characteristics

Designed by the researchers, general information was gathered on gender, age, education level, residential location, marital status, employment status, religious affiliation, monthly per capita household income, other diseases, previous knowledge about PLC, source of information about PLC and family history of PLC.

#### Primary outcome

##### Decision conflict

Decisional Conflict Scale (DCS) was developed by Professor O' Connor [20], and the main value was to assess the patient’s perception of decision uncertainty (Cronbach’s α 0.78–0.92). The DCS consists of 3 subscales: (1) uncertainty about choosing among different options, (2) modifiable factors that cause this uncertainty, and (3) perceived effectiveness of the choice. With a total of 16 items, the total scores range from 0 (no decision conflict) to 100 (highest-level in decision conflict), using the Likert 5-level scoring (ranging from 0 to 4). In the present study, we evaluated decision conflict using the modified Chinese version of DCS created by Lu et al. [21] at pre- and post-interventions. The Chinese version demonstrated a high internal consistency (Cronbach’s α 0.886).

#### Secondary outcomes

**(1) Decision preparation** Decision preparation referred to patients’ preparation for the choices they would face after receiving decision aids or decision support interventions, which was usually evaluated by Preparation Decision Making (PrepDM) Scale (Cronbach’s α 0.92–0.96) [22]. The total 10 items of the scale were scored on the Likert 5-point scale (ranging from 1 to 5) with a total score of 0–100. The higher the score, the better the decision preparation and the more effective the decision aids. This research evaluated decision preparation using the Chinese version of PrepDM Scale created by Li Yu et al. [7] at pre- and post-interventions. This Chinese version demonstrated a high internal consistency (Cronbach’s α 0.946).

**(2) Decision self-efficacy** Decision self-efficacy is the “self-confidence or belief in one’s abilities in decision making, including shared decision making”. Participants’ decision self-efficacy was assessed at pre- and post-interventions using an adapted version of O’Connor’s 11-item Decision Self-Efficacy Scale (DSES) (Cronbach’s α 0.899) [23]. Response options were measured on a 5-point Likert scale ranging from 0 (not at all confident) to 4 (very confident). Based on O’Connor’s user guide, the total self-efficacy score was created by summing the 11 items, divided by 11 and then multiplied by 25. The total score ranges from 0 (not confident) to 100 (extremely confident). This research evaluated decision self-efficacy using the Chinese version of DSES created by Wang Sitong et al. [24].

**(3) Satisfaction with decision-making** Participants’ decision satisfaction was assessed at post-intervention using the Satisfaction with Decision(SWD) Scale compiled by Holmes-Rovner (Cronbach’s α 0.860) [25], with a total of 6 items, using the 5-point Likert scale ranging from 1 (strongly disagree) to 5 (strongly agree). The total score ranges from 0 (not satisfied) to 30 (extremely satisfied). The SWD scale was to address a problem in the literature on patients’ satisfaction and desire to participate in decision-making.

**(4) Knowledge of PLC treatment** Participants’ knowledge of PLC treatment was assessed at pre- and post-interventions using 17 items in reference to Breast Cancer Knowledge Scale (Cronbach’s α 0.551) [26]. Knowledge domains included liver function, PLC risk factors, PLC screening, and PLC symptoms. Response options for each item were “true”, “false”, or “I don’t know”. A total knowledge score was calculated by summing the 17 items for purposes of analysis (1 point for each correct item with a possible total score of 17).

**(5) Decision regret** Participants’ decision regret was assessed by Decision Regret Scale (DRS) (Cronbach’s α 0.81 to 0.92) [27] at 3-month, with a total of 5 items, using the 5-point Likert scale ranging from 1 (strongly agree) to 5 (strongly disagree). Scoring involved reversing the scores of the 2 negatively phrased items, then taking the average of the 5 items. These averages were converted to a score ranging from 0 (do not regret at all) to 100 (extremely regret) by subtracting 1 and multiplying by 25.

### Statistical analysis

Data analysis was performed using SPSS (version 22.0). Mean, Standard Deviation, Frequencies and Percentages were used to describe the basic characteristics of PLC patients and study variables. To determine if the data were normally distributed, a histogram was used to illustrate the distribution. The baseline characteristics and outcomes of the participants were compared between patients in the two groups using the independent t-test or Wilcoxon test. In addition, paired t-test or Wilcoxon test was utilized for within-group comparisons before and after the intervention. Confidence interval was 95%. A two-tailed *P* value of < 0.05 was considered statistically significant.

## Results

### Patient characteristics

The mean age of the participants was 51.7 (8.39) in the control group and 50.0 (9.03) in the intervention group. There were no significant differences between the control and intervention group in socio-demographic data, previous knowledge about PLC, and source of knowledge (Table 1).

**Table 1:** Patient Characteristics (n = 180)

Characteristics	Control (n = 90) n (%)	Intervention(n = 90) n (%)	Total n (%)	*P*-value*
*Age (mean (SD))*	51.7(8.39)	50.0(9.03)	50.6(9.44)	0.134
*Gender*				
Male	71 (78.9)	68 (75.6)	139 (77.2)	0.594
Female	19 (21.1)	22 (24.4)	41 (22.8)	
*Marital status*				
Married	64 (71.1)	69 (76.7)	133 (73.9)	0.396
Others	26 (28.9)	21 (23.3)	47 (26.1)	
*Residential location*				
City	51 (56.7)	61 (67.8)	112 (62.2)	0.124
Village	39 (43.3)	29 (32.2)	68 (37.8)	
*Education level*				
Primary school	16 (17.8)	10 (11.1)	26 (14.4)	0.187
Junior high school	33 (36.7)	26 (28.9)	59 (32.8)	
Senior high school	28 (31.1)	32 (35.6)	60 (33.3)	
College or above	13 (14.4)	22 (24.4)	35 (19.5)	
*Working status*				
Employed	61 (67.8)	71 (78.9)	132 (73.3)	0.092
Others	29 (32.2)	19 (21.1)	48 (26.7)	
*Household's average monthly income*				
< 1000 yuan (About USD160)	10 (11.1)	6 (6.7)	16 (8.9)	0.429
1000–3000 yuan	48 (53.3)	42 (46.7)	90 (50.0)	
3000–5000 yuan	24 (26.7)	31 (34.4)	55 (30.6)	
> 5000 yuan	8 (8.9)	11 (12.2)	19 (10.6)	
*Religious affiliation*				
Yes	16 (17.8)	11 (12.2)	27 (15.0)	0.297
No	74 (82.2)	79 (87.8)	153 (85.0)	
*Personality*				
Extrovert	48 (53.3)	56 (62.2)	104 (57.8)	0.227
Introvert	42 (46.7)	34 (37.8)	76 (42.2)	
*With other diseases*				
No	13 (14.5)	15 (16.7)	28 (15.6)	0.222
1–2 type	56 (62.2)	63 (70.0)	119 (66.1)	
More than 2 types	21 (23.3)	12 (13.3)	33 (18.3)	
*Previous knowledge about PLC*				
Low	44 (48.9)	51 (56.7)	95 (52.8)	0.562
Moderate	38 (42.2)	33 (36.7)	71 (39.4)	
High	8 (8.9)	6 (6.6)	14 (7.8)	
*Source of knowledge about PLC*				
Study	21 (23.3)	17 (18.9)	38 (21.1)	0.514
Health staff	10 (11.1)	16 (17.8)	26 (14.4)	
Mass media	54 (60.0)	50 (55.6)	104 (57.8)	
Acquaintances/Friends	5 (5.6)	7 (7.8)	12 (6.7)	
*Family history of PLC*				
Parents	63 (70.0)	50 (55.6)	113 (62.8)	0.174
Others	20 (22.2)	30 (33.3)	50 (27.8)	
No family history	7 (7.8)	10 (11.1)	17 (9.4)	

This table shows that the two groups were homogeneous in terms of demographic characteristics, including age, gender, marital status, residential location, education level, working status, household's average monthly income, religious affiliation, personality, as well as with other diseases, previous knowledge about PLC, source of knowledge about PLC, and family history of PLC.

*Chi-square test

### Results of the two groups before intervention

The mean score differences in all aspects before the intervention of the two groups are shown in Tables 2 and 3. There were no significant differences between the control and intervention group in decision conflict, decision preparation, decision self-efficacy and knowledge of PLC treatment.

**Table 2:** Comparison of the mean scores of the primary outcomes in the two groups

Variable	Time	Control (n = 90) Mean ± SD	Intervention (n = 90) Mean ± SD	*P*-value*
Decision conflict	Before the intervention	29.79 ± 9.22	28.44 ± 11.34	0.381*
	After the intervention	26.75 ± 9.79	16.89 ± 8.80	0.001*
<subscale>				
Uncertainty	Before the intervention	5.95 ± 2.70	5.24 ± 2.61	0.054**
	After the intervention	5.61 ± 2.69	2.73 ± 2.44	0.001**
Uncertainty factors	Before the intervention	17.58 ± 5.84	16.48 ± 6.96	0.248*
	After the intervention	13.66 ± 6.01	9.91 ± 5.81	0.001*
Clarity***	Before the intervention	6.25 ± 2.91	7.01 ± 3.43	0.095**
	After the intervention	7.48 ± 3.37	4.25 ± 3.40	0.001**

*Independent t-test, **Mann–Whitney test, ***A lower clarity score indicates less decisional conflict

**Table 3:** Comparison of the mean scores of the secondary outcomes in the two groups

Variable	Time	Control (n = 90) Mean ± SD	Intervention (n = 90) Mean ± SD	*P*-value*
Decision preparation	Before the intervention	55.42 ± 7.93	57.07 ± 7.56	0.156*
	After the intervention	63.84 ± 7.38	80.73 ± 8.16	0.001*
Decision self-efficacy	Before the intervention	74.04 ± 14.04	76.57 ± 10.96	0.180*
	After the intervention	76.89 ± 13.46	87.75 ± 6.87	0.001*
Knowledge of PLC	Before the intervention	9.48 ± 2.90	10.02 ± 2.92	0.211*
	After the intervention	12.72 ± 2.13	14.52 ± 1.91	0.001**
Satisfaction with decision-making	After the intervention	23.12 ± 3.91	25.68 ± 2.10	0.001*
Decision regret	Three month after the intervention	32.33 ± 9.22	30.00 ± 12.06	0.070**

*Independent t-test, **Mann–Whitney test

### Results of the two groups after intervention

#### Primary outcome

The mean score differences in the primary outcomes between the intervention and control groups are shown in Table 2. The test showed that statistically the control group had a significantly higher DCS score than the intervention group after the intervention (intervention group: 16.89 ± 8.80 vs. control group: 26.75 ± 9.79, *P* < 0.05).

#### Secondary outcomes

**Fig. 2:**
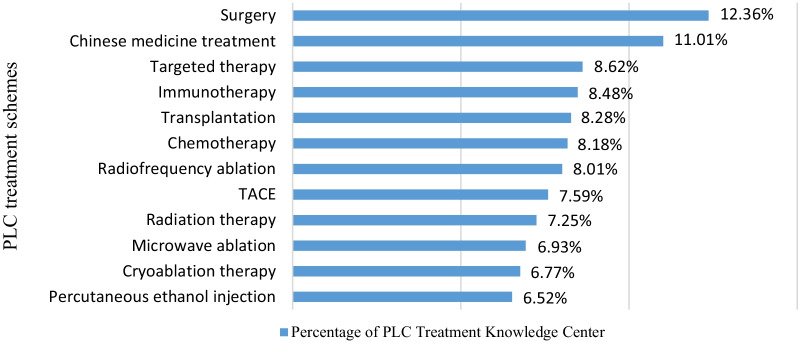
The browsing frequency of PLC Treatment Knowledge Center in the intervention group, after intervention

The mean score differences in the secondary outcomes in the intervention and control groups are shown in Table 3. The research showed that the intervention group had a significantly higher preparation, self-efficacy and satisfaction score in decision-making than the control group (*P* < 0.05). Also, the test showed that the intervention group had a significantly higher knowledge score (14.52 ± 1.91) than the control group (12.72 ± 2.13), (*P* < 0.05). At 3-month follow-up, we did not find significant difference in decision regret between the two groups (*P* > 0.05). Figure 2 shows the frequency of patients in the intervention group browsing each knowledge module of PLC Treatment Knowledge Center after intervention. According to the results, the highest frequency was related to surgery (12.36%) and traditional Chinese medicine treatment (11.01%).

### Results of the intervention group before and after intervention

The mean score differences in the outcomes of the intervention group are shown in Table 4. There were significant differences between the scores of decision conflict, decision preparation, decision self-efficacy and knowledge score before and after intervention (*P* < 0.05).

**Table 4:** Comparison of the mean scores of the two groups before and after the intervention

Variable	Before the intervention	After the intervention	*P*-value*
*Intervention group*			
Decision conflict	28.44 ± 11.34	16.89 ± 8.80	0.001*
Decision preparation	57.07 ± 7.56	80.73 ± 8.16	0.001*
Decision self-efficacy	76.57 ± 10.96	87.75 ± 6.87	0.001*
Knowledge of PLC	10.02 ± 2.92	14.52 ± 1.91	0.001**
*Control group*			
Decision conflict	29.79 ± 9.22	26.75 ± 9.79	0.028*
Decision preparation	55.42 ± 7.93	63.84 ± 7.38	0.001*
Decision self-efficacy	74.04 ± 14.04	76.89 ± 13.46	0.113*
Knowledge of PLC	9.48 ± 2.90	12.72 ± 2.13	0.001*

*Paired t-test, **Wilcoxon test

### Results of the control group before and after intervention

The mean score differences in the outcomes of the control group are also shown in Table 4. There was some difference in the scores of decision conflict, decision preparation and knowledge score before and after intervention (*P* < 0.05), but there was no significant difference in the level of decision self-efficacy (*P* = 0.113).

## Discussion

This study primarily evaluated the quality of patients’ decision-making in shared decision-making from six aspects: decision conflict, decision preparation, decision self-efficacy, decision satisfaction, decision regret and knowledge of PLC treatment.

### Decision conflict

It is generally believed when patients are faced with two or more treatment options or examinations, they may encounter uncertainty or hesitation to make decisions in accordance with their own conditions and value preferences, hence subsequent decision conflict [21]. Our results showed a significantly lower decision conflict in intervention group compared to control group, in line with results of a recent Cochrane Library meta-analysis of 63 randomized controlled trials [28]. This may be explained by three reasons. First of all, “SDM Assistant” provided evidence-based information support, which was easily accessible for PLC patients through smartphones; it seemed to satisfy patients’ need for PLC treatment knowledge and most effectively stimulated patients’ interest in reading. Then, the “Decision Aids Path” of the app was an established logical route to structurally guide patients through their decision-making following their own preferences, which improved patients’ willing to utilize the app. Moreover, compared to traditional decision aids that present detailed decision information only by text descriptions, the “SDM Assistant” contains diversified means of expression to empower patients to intuitively understand decision information [19]. A study by Hochlehnert et al. [29] showed a significantly lower decision conflict and a higher decision satisfaction among patients with fibromyalgia syndrome who benefited from the decision aids based on website. Bernard et al. [18] constructed a breast reconstruction learning module based on e-Health in order to alleviate the doubts about breast reconstruction surgery in breast cancer women in the United States. These procedures increased patients’ participation and reduced uncertainty in decision-making, as well as achieved better therapeutic effects, which were in line with our study.

### Preparation, self-efficacy, satisfaction and regret with decision-making

Decision preparation refers to the readiness of individuals to face choices after receiving decision aids [22]. In this study, the “SDM Assistant” largely improved decision-making preparation among the participants in intervention group. For instance, it made the patients better understand PLC treatment, and also made them more active in their own decision-making. Similarly, a series of research on various types of diseases, such as breast cancer [30], prostate cancer [31], and other diseases [28], showed that the decision aids application providing information on the benefits and risks can enhance patients’ decision-making preparation.

Informed decision-making is a process in which individuals understand the information of a disease and choose treatment options according to their personal values [32]. Previous studies [33, 34] have shown that the level of patients’ decision self-efficacy was an important factor to promote patients in finalizing their selections. In the present study, the “SDM Assistant” intervention promoted the PLC patients’ self-efficacy in decision-making. Enhancement of the patients’ self-efficacy indicated that the “SDM Assistant” had assured the patients that they had the ability in decision-making. Therefore, they were more willing to cooperate with medical and nursing work. In other words, the well-known KABP model is verified, namely, PLC treatment knowledge can improve the level of decision-making self-efficacy of patients, and then promote patients' participation in treatment decision-making. An American study [35] in Spanish American male patients with prostate cancer who were given web-based decision aids, also confirmed that the web decision aids significantly improved the decision-making self-efficacy level of patients and made them more confident in treatment. In addition, studies had shown that adequate emotional support would also bring patients more confidence in treatment options. Therefore, “SDM Assistant” had incorporated a “family participation mode” and encouraged family members to participate in decision-making together with patients.

At present, many studies have confirmed that decision aids can improve the relationship between doctors and patients, thus improving the satisfaction of patients with the treatment process [36–38]. The present study findings revealed an improvement of participants’ decision satisfaction in the intervention group compared to the control group. The result was consistent with the conclusion of a systematic review using decision aids in different situations [28]. In addition, the “SDM Assistant” was a smartphone application, which was easily accessible and could be browsed by patients, regardless of time and place. As a result, it improved the participants’ experience as well as their satisfaction.

In the present study, patients from the two groups were followed up for 3 months after discharge. It was found that there was almost no difference in decision regret scores between the two groups. This might be explained by the short follow-up time, for patients were still in the process of rehabilitation, so they could not judge the correctness of the decision. In the future, further research will continue to add the follow-up evaluation function and set to evaluate the decision regret and satisfaction every 3 months to obtain long-term results.

The study findings revealed an increase in the knowledge level of PLC after the intervention. Cochrane Library, Stacy et al. in 2017 [39] did a systematic review that covered 105 studies on decision aids, of which 71 (67.6%) evaluated the impact of decision aids on patients’ knowledge level. The test content was based on the information support in decision aids. Among the 71 studies, 52 studies (high-quality evidence) showed that providing decision aids for patients was better than usual care in improving patients’ knowledge level. Based on “SDM Assistant”, the decision aids transfers the PLC treatment information to patients in plain language. Thus, it aroused patients' interest in seeking information. At the same time, it provides patients with a path to acquire knowledge and promotes patients' behavior of acquiring knowledge.

In addition, due to the outbreak of COVID-19, it is rather inconvenient for patients to go to the hospital for review or face-to-face health education. However, the app will not be restricted by time or place. In this context, the app can help patients understand PLC treatment knowledge and clear their own values right on a timely basis. That may contribute to their effective communication with their physicians and active participation in decision-making. Therefore, such applications are especially valuable when the world is fighting against the COVID-19 pandemic.

## Limitations

As far as we know, this is the first study in China to assist PLC patients with “SDM Assistant” to participate in treatment options. However, some limitations need to be considered. First, the participants recruited in this study were all with high health literacy and education levels, being skilled in using electronic products, and active in participating in decision-making, so their compliance was higher, and accordingly their decision-making participation and outcomes were better. What worries us is that for elderly patients and those with lower health literacy, such as those in rural or remote areas, we are not sure whether they will use the app and accurately understand the content and expression of the app. Second, we did not include the factors of patients as an influencing factor in this study. We plan to recruit PLC patients with different levels of education and health literacy to promote the App in future studies, and at the same time continuously optimize and improve the SDM App by further exploring the factors influencing its usability. Third, our study supports the use of interactive decision aids to help patients make the treatment decisions according to their values. However, this app is still in the first stage of research and development with single functions and simple forms of expression. Future work will evaluate whether the content and form of decision aids are important, and improve the quality of decision aids to enrich the app features and content. Besides, as a result of the outbreak of Covid-19, most of the patients couldn’t manage to come to our hospital for a check-up 3 months after discharge, which greatly limited the information we could collect.

What’s more, although the baseline of the two groups of patients is comparable, we cannot determine whether the participants have obtained decision-making knowledge from other information channels during the intervention. Since patients’ post-intervention knowledge will depend on the information provided to them (either by the medical staff or the app), if there are differences between the baseline information they receive in both conditions, their level of knowledge cannot inform us about the actual effectiveness of the app on improvements in knowledge. Further studies should address this problem accordingly. It’s also important to note that the participants of this study are all informed PLC patients in China. Due to differences in language, culture and medical systems in various countries, the results may not be universal. Finally, as the research was conducted in the hospital via a smartphone application, it is impossible to double-blind the researchers (doctors, nurses) and the PLC patients. Therefore, this study is a quasi-experimental study and the deviation of the results cannot be completely eliminated. Next, we will consider a randomized controlled trial to reduce the bias of the results.

## Conclusions

Against the background of the gap between PLC patients’ needs in SDM and a shortage of medical resources in China, this study demonstrated that the smartphone based application “SDM Assistant” could decrease the patients’ decision conflict, promote the decision preparation, self-efficacy, satisfaction and PLC knowledge level. Therefore, promotional activities with “SDM Assistant” are recommended to improve informed PLC patients’ performance in the field of decision-making, and strengthen the relationship between physicians and patients, especially in remote regions during the COVID-19 pandemic. Moreover, it is necessary to update and manage the “SDM Assistant” to guarantee that the content is based on best evidence available, acknowledges areas of uncertainty, and provides probabilities of various real outcomes. Via the use of “SDM Assistant”, we are committed to realizing “I Choose My Own Treatment”.

## Data Availability

The datasets generated and analyzed during the current study are available upon reasonable request. Applicants can send their request for study data to Dr. Li via email: smallsweetlily@163.com.

## Abbreviations

PLC: Primary liver cancer
SPSS: Statistical package for the social sciences
CNCC: Chinese National Cancer Center
BCLC: Barcelona Clinic Liver Cancer
SDM: Shared Decision-Making
DAs: Decision Aids
SDM Assistant: Shared Decision Making Assistant
ODSF: Ottawa Decision Support Framework
SD: Standard Deviation
DCS: Decisional Conflict Scale
PrepDM: Preparation Decision Making
DSES: Decision Self-Efficacy Scale
SWD: Satisfaction with Decision
DRS: Decision Regret Scale
